# Clomiphene Citrate Ameliorates Hyperglycemic Phenotype Induced by Catch-Up Growth in Stunting-Like *Drosophila*

**DOI:** 10.1021/acsomega.5c12117

**Published:** 2026-04-07

**Authors:** Hendra Stevani, Habibie Habibie, Muhammad Rayza Azmin, Nadila Pratiwi Latada, Asbah Asbah, Widya Hardiyanti, Mukarram Mudjahid, Muh. Arfandy Gunawan, Firzan Nainu

**Affiliations:** † Doctoral Program in Pharmacy, Faculty of Pharmacy, 64739 Hasanuddin University, Tamalanrea, Makassar 90245, Indonesia; ‡ Program Study of Bachelor of Applied Pharmacy, Department of Pharmacy, Health Polytechnic of Makassar, Ministry of Health of the Republic of Indonesia, Baji Gau, Makassar 90134, Indonesia; § Department of Pharmacy, Faculty of Pharmacy, Hasanuddin University, Tamalanrea, Makassar 90245, Indonesia; ∥ Unhas Fly Research Group, Faculty of Pharmacy, Hasanuddin University, Tamalanrea, Makassar 90245, Indonesia; ⊥ Program Study of Pharmacy, Faculty of Medicine and Health Sciences, Universitas Muhammadiyah Makassar, Makassar 90221, Indonesia; # Chemical Biology and Medicinal Chemistry Research Group, Faculty of Pharmacy, Hasanuddin University, Makassar 90245, Indonesia

## Abstract

Catch-up growth following early life undernutrition is a major risk factor for metabolic dysfunction, including insulin resistance and type 2 diabetes mellitus. Here, we developed a *Drosophila melanogaster* model of catch-up-growth-associated metabolic dysregulation and used clomiphene citrate (CC) as a pharmacological probe to explore conserved metabolic regulatory pathways. Larvae were maintained on a low-calorie diet until the early third instar and subsequently returned to a standard diet to induce compensatory growth. Metabolic phenotyping included glucose levels, reactive oxygen species (ROS), triglycerides, locomotor performance, and transcriptional analysis of insulin- and hormone-related regulators (*dilp2, dInR, dPHM*, and *dHNF4*). Catch-up growth flies exhibited sustained hyperglycemia, elevated ROS, and increased triglyceride levels, indicating dysregulated glucose and lipid homeostasis, while locomotor function remained relatively preserved at the early adult stages. CC supplementation was associated with attenuation of hyperglycemia and oxidative stress, together with modulation of insulin- and hormone-related transcriptional markers. Molecular docking and molecular dynamics simulations provided hypothesis-generating structural insights into potential interactions of CC with metabolic regulators. Collectively, these findings establish *Drosophila* as a tractable model for investigating early-stage metabolic consequences of catch-up growth and support the use of CC as a tool compound for mechanistic exploration, while highlighting the need for further validation in mammalian systems.

## Graphical abstract

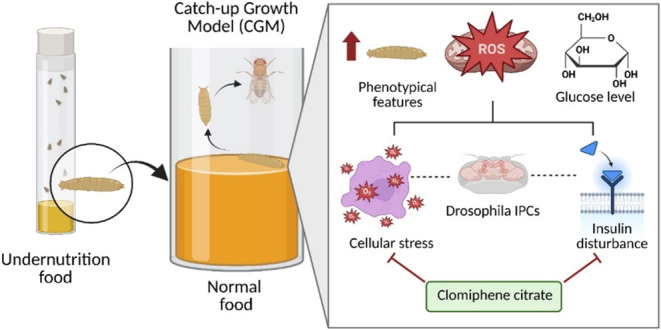

## 1 Introduction

Catch-up growth is an essential compensatory process in infants born with low birth weight, enabling recovery from early nutritional deficits and supporting the attainment of normal organ development.^1^ Despite its physiological importance, this accelerated postnatal growth trajectory has been consistently associated with increased susceptibility to metabolic disorders in later life, including insulin resistance and diabetes mellitus.^2–4^ These risks highlight the need for effective strategies that allow children to benefit from catch-up growth while mitigating its long-term metabolic consequences, including approaches such as drug repurposing.

One of the major challenges in drug repurposing using biological experimental approaches is the selection of an appropriate animal model. In particular, an experimental model suited for evaluating repurposed drugs to prevent diabetes mellitus under catch-up growth conditions has not yet been established, making model selection a distinct methodological obstacle. Computational strategies have also been applied to drug repurposing for type 2 diabetes mellitus (T2DM). For example, a transformer-based deep learning framework identified several promising candidate compounds, including triterpenes, Sho-saiko-to, LY294002, mitogen-activated protein kinase (MAPK) inhibitors, and clomiphene citrate (CC).^5^

Emerging evidence shows that children born with low birth weight who subsequently undergo catch-up growth exhibit reduced sex hormone-binding globulin (SHBG) levels and elevated androgen concentrations-endocrine alterations that are strongly associated with insulin resistance and an increased risk of T2DM.^6^ SHBG has further been characterized as a biomarker linking sex-hormone dysregulation to adverse metabolic profiles across both pediatric and adult populations.^7^ These findings suggest that disruption of sex-hormone homeostasis constitutes an important endocrine feature linking catch-up growth to later-life metabolic vulnerability. CC is a selective estrogen receptor modulator capable of increasing endogenous testosterone and modulating the sex-hormone dynamics. However, in the present study, CC is not positioned as a conventional antidiabetic agent. Rather, CC is employed as a compound to interrogate whether modulation of sex-hormone-related pathways during critical developmental windows can influence long-term metabolic programming following catch-up growth. Although clinical data regarding CC’s direct influence on glucose-insulin homeostasis remain preliminary,^8^ its ability to modify hormonal dynamics and its reported favorable metabolic signals in specific dysmetabolic populations support its consideration as a repurposing candidate within this experimental framework.

The fruit fly (*Drosophila melanogaster*) is widely recognized as a versatile model organism in biomedical research due to its highly conserved genetic architecture, short life cycle, and tractable experimental system. Recent reviews highlight that *D. melanogaster* shares a substantial proportion of its genome with humans, supporting its relevance for investigating fundamental biological processes and mechanisms underlying human diseases.^9^ Moreover, complementary evidence indicates that up to 75% of human disease-associated genes have identifiable orthologues in *Drosophila*, underscoring its strong translational value for modeling disease pathways and preclinical drug discovery.^10^ The species has also been widely employed in studies of T2DM, particularly for investigating insulin resistance, lipid dysregulation, and diet-induced metabolic imbalance.^11–13^ Nevertheless, it is important to note that *D. melanogaster* lacks canonical mammalian estrogen receptors, indicating that any metabolic effects of CC observed in this model are unlikely to be mediated through classical estrogen receptor signaling. Instead, CC is hypothesized to act via estrogen-receptor-independent mechanisms converging on conserved metabolic pathways, such as insulin/IGF signaling and lipid metabolism. In addition, *Drosophila* is easy to maintain, has a short developmental cycle, and is highly cost-effective compared with vertebrate models.^14^ Building on these advantages, the present study sought to establish a *D. melanogaster* catch-up growth model that recapitulates key metabolic and molecular features of hyperglycemia and to use this model to evaluate the mechanistic and phenotypic effects of CC through phenotypic and gene-expression-based analyses.

## 2 Materials and Methods

### 2.1 Materials

Sucrose (CAS No: 57–50–1, Smart Lab, Indonesia). Clomiphene citrate (CAS No: 50–41–9, Nanjing Duly Biotech, China) was used as a candidate for drug repurposing.

### 2.2 Drosophila Stock

Wild-type *D. melanogaster* strain *Oregon-R­(R)* was obtained from the Kyoto *Drosophila* Stock Center (Kyoto Institute of Technology, Kyoto, Japan) and subsequently maintained at the Laboratory of Pharmacology and Toxicology, Faculty of Pharmacy, Hasanuddin University, Indonesia. The flies were reared under controlled conditions at 25 °C with 60% humidity and a 12 h light/dark cycle.

### 2.3 Preparation of Drosophila Diet

Four formulations of the *D. melanogaster* diet were prepared, and the detailed composition of each is provided in Table 1.

**Table 1:** Composition of *Drosophila* Feed

composition	standard diet	low-calorie diet (7)	standard diet + clomiphene citrate 0.1 mM	standard diet + clomiphene citrate 0.5 mM
Corn Flour (g)	7.5	1.25	7.5	7.5
Yeast (g)	2.5	0.3125	2.5	2.5
Agar (g)	0.9	1.5	0.9	0.9
Sucrose (g)	4.5	0.75	4.5	4.5
Glucose (g)	-	1.5	-	-
Propionic acid (μL)	400	400	400	400
Methyl parabens (μL)	450	450	450	450
EtOH 70% (μL)	-	-	7.5	7.5
Clomiphene citrate (mM)	-	-	0.1	0.5
Water up to (mL)	100	100	100	100

A low-calorie diet was formulated to induce a stunting-like phenotype in *Drosophila*, thereby establishing the nutritional conditions necessary for subsequent catch-up growth. CC was repurposed as a preventive intervention targeting T2DM-related metabolic disturbances that arise from this growth trajectory. For supplementation, CC was dissolved in 70% ethanol and incorporated into the standard diet to yield final concentrations of 0.1 and 0.5 mM. Independent laboratory proximate analysis confirmed that the low-calorie diet contained substantially lower total energy than the standard diet (18.69 vs 66.4 kcal/100 g), with correspondingly reduced carbohydrate, protein, and lipid fractions. Detailed caloric and macronutrient composition of the experimental diets is provided in Table S1.

### 2.4 Protocol for Producing Catch-Up Growth Flies

Adult flies were first allowed to mate and oviposit on a low-calorie diet, ensuring that the resulting offspring developed under nutrient-restricted conditions until reaching the early third-instar stage. At this point, larvae assigned to the growth-chased group were transferred to a standard diet to induce compensatory growth through maturation into adulthood. In parallel, undernourished control flies remained on the low-calorie diet throughout development, providing a critical comparator for assessing the metabolic consequences of catch-up growth. For the treatment groups, early third-instar larvae were transferred to a standard diet supplemented with clomiphene citrate at final concentrations of 0.1 or 0.5 mM, enabling evaluation of the compound’s preventive potential under catch-up growth conditions. In addition, metformin (25 mM)^15^ was included as a positive control and administered via the standard diet to a separate catch-up growth group to validate the responsiveness of the model to a known antihyperglycemic intervention. A schematic overview of the experimental procedures is presented in Figure 1.

**Figure 1:**
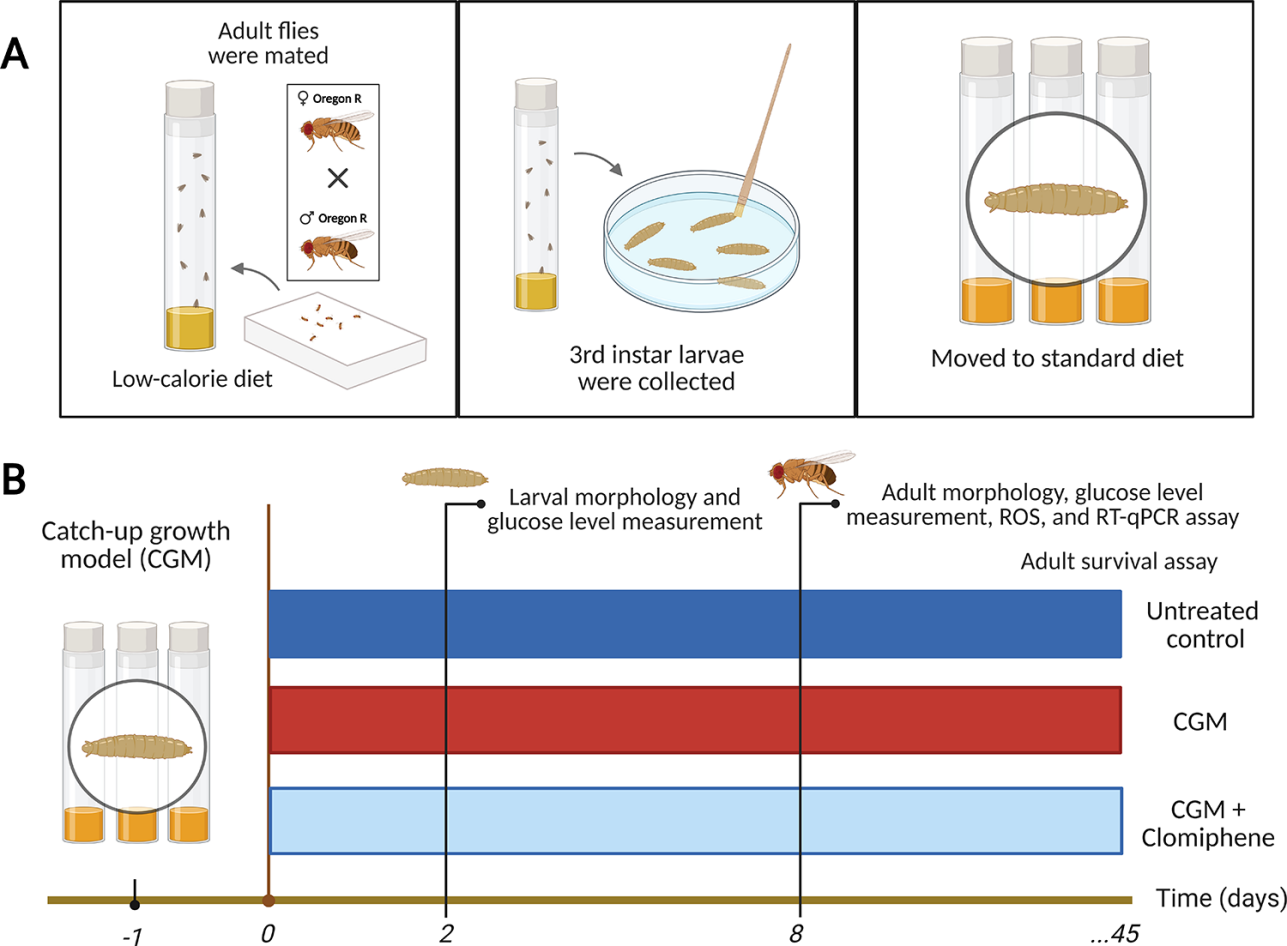
Schematic overview of the experimental workflow. (A) Establishment of the catch-up growth model (CGM). (B) Chronological timeline outlining the major experimental procedures. Created in BioRender. Nainu, F. (2026) https://BioRender.com/n7pqtu2.

### 2.5 Comprehensive Measurements of Body Weight and Surface Area

Body weight was determined by weighing batches of 20 late third-instar larvae using an analytical balance, and the average weight was calculated for accuracy. Larval length and adult surface area were quantified using light microscopy combined with ImageJ analysis. Measurements were obtained from 10 larvae and 10 adult flies per group, and average values were used to ensure reliability and consistency in assessing growth patterns.

### 2.6 Survival Assay

The survival assay was conducted following previously published protocols,^15^ with several modifications, to evaluate developmental progression from the larval stage through pupation and eclosion. Third-instar larvae were transferred into vials containing their designated diets, while control groups received standard feed, whereas treatment groups were provided with diets supplemented with 0.1 or 0.5 mM CC. Each vial contained 10 larvae. All experiments were performed at 25 °C, and food was replaced every 3 days. Larvae were monitored daily to document transitions from larva to pupa and from pupa to adult, enabling quantitative assessment of developmental success across groups.

### 2.7 Measurement of Glucose Levels in Larvae

Measurement of glucose level in larvae was performed following a previously published protocol,^16^ with several modifications. Briefly, 80 third-instar larvae were collected and transferred into a microcentrifuge tube and then homogenized thoroughly using a micropestle. The homogenate was centrifuged at 13,000 rpm for 1 min, after which 10 μL of the resulting supernatant was transferred into a fresh 1.5 mL microcentrifuge tube. Subsequently, 1000 μL of the glucose oxidase-peroxidase aminoantipyrine (GOD-PAP) reagent (Glory Diagnostics, Barcelona, Spain) was added. The mixture was incubated at 37 °C for 10 min, and absorbance was measured at 500 nm using a SAFAS UVmc1 spectrophotometer (SAFAS, Monaco, France). A glucose standard solution (100 mg/dL) served as the reference for the quantification.

### 2.8 Measurement of Glucose and Triglyceride Levels in Adult Flies

Glucose and triglyceride levels in adult *D. melanogaster* were measured following previously published protocols^17,18^ with minor modifications. Briefly, ten adult flies per biological replicate were collected, anesthetized by brief cooling in a refrigerator, and transferred into a microcentrifuge tube. Flies were homogenized using a micropestle, after which 200 μL of phosphate-buffered saline (PBS, pH 7.4) was added. The homogenate was centrifuged at 13,000 rpm for 7 min, heated at 70 °C for 5 min to inactivate endogenous enzymes, and centrifuged again at 13,000 rpm for 15 min. For glucose measurement, a 10 μL aliquot of the resulting supernatant was mixed with 1000 μL of GOD-PAP reagent (Glory Diagnostics, Barcelona, Spain) and incubated at 37 °C for 10 min. Absorbance was measured at 500 nm by using a SAFAS UVmc1 spectrophotometer (Monaco, France). For triglyceride measurement, a separate 10 μL aliquot of the supernatant was combined with 1000 μL of glycerol phosphate oxidase-peroxidase aminoantipyrine (GPO-PAP) reagent (Glory Diagnostics, Barcelona, Spain) and incubated at 37 °C for 10 min. Absorbance was measured at 500 nm using the same spectrophotometer. Quantification of glucose and triglyceride concentrations was performed by using manufacturer-provided standard solutions, and values were normalized per group of flies.

### 2.9 Measurement of ROS Levels

Reactive oxygen species (ROS) levels in the hemolymph of adult flies were quantified using a nitro blue tetrazolium (NBT) reduction assay conducted according to a previously published protocol.^16^ A total of 100 adult flies were collected and rinsed with PBS to remove residual food. Hemolymph was extracted on ice to minimize the level of melanization. A final volume of 300 μL was prepared by mixing 100 μL of hemolymph with 200 μL of 1× PBS, followed by the addition of an equal volume of the NBT solution. The mixture was incubated at room temperature in the dark for 1 h, after which 300 μL of 100% glacial acetic acid was added to terminate the reaction. Following centrifugation at a maximum speed for 1 min, absorbance was measured at 595 nm after the addition of 50% acetic acid.

### 2.10 Locomotor Assay

Locomotor performance was evaluated by using a negative geotaxis (climbing) assay. Groups of 10 adult male flies were placed in a vertical tube and gently tapped to the bottom. The percentage of flies that climbed above 8 cm within 15 s was recorded as the climbing index. Each condition was tested in three independent biological replicates with three technical repeats per replicate.^19^

### 2.11 Gene Expression Analysis

This procedure was performed following a previously published protocol.^20^ Ten larvae from each treatment group were subjected to RNA isolation using the Monarch Total RNA Miniprep Kit (New England Biolabs, Inc., MA), following the manufacturer’s instructions. Gene expression levels were quantified using the Luna Universal RT-qPCR Kit (New England Biolabs, Inc., MA) following the recommended protocol. RT-qPCR reactions were prepared in a total volume of 10 μL, and amplification of target genes (primer sequences listed in Table 2) was carried out using the Rotor-Gene Q system (Qiagen, Germany).

**Table 2:** Primers Used in the RT-qPCR Assay

Gene	Forward primer (5′-3′)	Reverse primer (5′-3′)	Refs
*dilp2*	TCTGCAGTGAAAAGCTCAACGA	CAAACTGCAGGGGATTGAGG	^21^
*dInR*	AAGCGTGGGAAAATTAAGATGGA	GGCTGTCAACTGCTTCTACTG	^22^
*dHNF4*	ACAACAACAGCATGTTCTCACC	TCGCCCGATCTCCACAAATG	-
*dPHM*	GGATTTCTTTCGGCGCGATGTG	TGCCTCAGTATCGAAAAGCCGT	^23^
*rp49*	GACGCTTCAAGGGACAGTATCTG	AAACGCGGTTCTGCATGAG	^20^

The *dHNF4* primer pair was obtained from FlyPrimerBank (DRSC/TRiP Functional Genomics Resources, Harvard Medical School; available at: https://www.flyrnai.org/flyprimerbank/) and subsequently validated in silico using NCBI BLAST to ensure target specificity and to exclude genomic off-target binding. The amplification procedure consisted of an initial cycle at 50 °C for 10 min, followed by 95 °C for 2 min, and then 35 cycles of 95 °C for 10 s, 60 °C for 30 s, and 72 °C for 30 s. Melt-curve and amplification analyses were performed to verify the specificity of the expected products. The *rp49* gene served as the internal control, and its expression was quantified using *rp49*-specific primers under the same thermal cycling conditions applied to the target genes.

### 2.12 Molecular Docking

The three-dimensional (3D) structures of *Drosophila* dilp2, the ecdysone receptor ligand-binding domain (EcR-LBD), and hepatocyte nuclear factor (HNF) were retrieved from the RCSB Protein Data Bank (http://www.rcsb.org/pdb). The structure of clomiphene citrate, used as the test ligand, was obtained from the PubChem database (https://pubchem.ncbi.nlm.nih.gov/).^18,24^ Prior to docking, all protein structures were prepared using UCSF Chimera by removing crystallographic ligands, adding polar hydrogen atoms, and assigning the appropriate protonation states. Molecular docking simulations were performed using AutoDock Vina implemented within UCSF Chimera, with grid box parameters positioned to encompass the predicted ligand-binding pockets of each target protein.^25^ The grid box coordinates and dimensions were defined as follows:

Dilp2: center = (216.07, 183.33, 230.52); size = (25.19 Å × 14.33 Å × 10.36 Å)

EcR: center = (61.44, 29.30, 12.90); size = (24.10 Å × 20.17 Å × 24.43 Å)

HNF: center = (64.61, 29.68, 11.73); size = (18.88 Å × 20.66 Å × 23.29 Å)

The resulting protein–ligand complexes were evaluated based on binding affinities (Δ*G*, kcal·mol^–1^) and molecular interactions, including hydrogen bonds and hydrophobic contacts, using Discovery Studio Visualizer.^26^

### 2.13 Molecular Dynamics Simulations

Molecular dynamics simulations were conducted using YASARA software to evaluate the interactions between clomiphene, the native ligands, and the target proteins Dilp2, EcR, and HNF. Simulations were performed using the AMBER14 force field under periodic boundary conditions at 310 K and pH 7.4, with TIP3P water molecules and counterions added to maintain system neutrality. A 100 ns production simulation was selected, as this time scale is commonly used to assess protein–ligand stability and equilibration in MD studies.^28^ Each system was subjected to a 100 ns simulation with a 0.25 fs time step. For each complex, a single continuous production run was performed. Structural stability and conformational dynamics were assessed by monitoring root-mean-square deviation (RMSD), root-mean-square fluctuation (RMSF), and radius of gyration (*R*_g_) at 25 ps intervals. Stabilization of RMSD and *R*_g_ over the simulation period was taken as indicative of adequate equilibration and internal stability of the complexes.^27^

### 2.14 Data Analysis

All data obtained from phenotypic and molecular assays were analyzed by using Prism 9 (*GraphPad Software, Boston, MA, USA*). Data are presented as bar graphs and expressed as mean ± standard deviation (mean ± SD). For comparisons involving more than two experimental groups, statistical significance was evaluated using one-way analysis of variance (ANOVA) followed multiple-comparison post hoc test. The number of independent biological replicates for each experiment is indicated in the corresponding figure legends. Statistical significance was defined as *p* < 0.05; exact *p* values for relevant group comparisons are reported in the corresponding figures and/or figure legends to allow transparent assessment of statistical differences among groups.

## 3 Results and Discussion

### 3.1 Feeding with a Low-Calorie Diet Followed by A Standard Diet-Induced Catch-Up Growth in Larvae

Growth retardation can result from pathological conditions or inadequate nutrient intake.^29^ In this study, catch-up growth was successfully induced by transferring nutrient-restricted larvae to a standard diet. Third-instar larvae reared initially on a low-calorie diet (Table 1) exhibited clear compensatory growth after dietary normalization, reaching body weights (Figure 2A) and lengths (Figure 2B) that were not significantly different from those of normally fed controls. In contrast, larvae maintained on a low-calorie diet throughout development showed a markedly reduced body size relative to the standard diet group. A comparable trend was observed in adults: growth parameters in the catch-up cohort were statistically indistinguishable from those of normally reared flies, indicating that compensatory growth persisted through metamorphosis and was effectively established across developmental stages.

**Figure 2:**
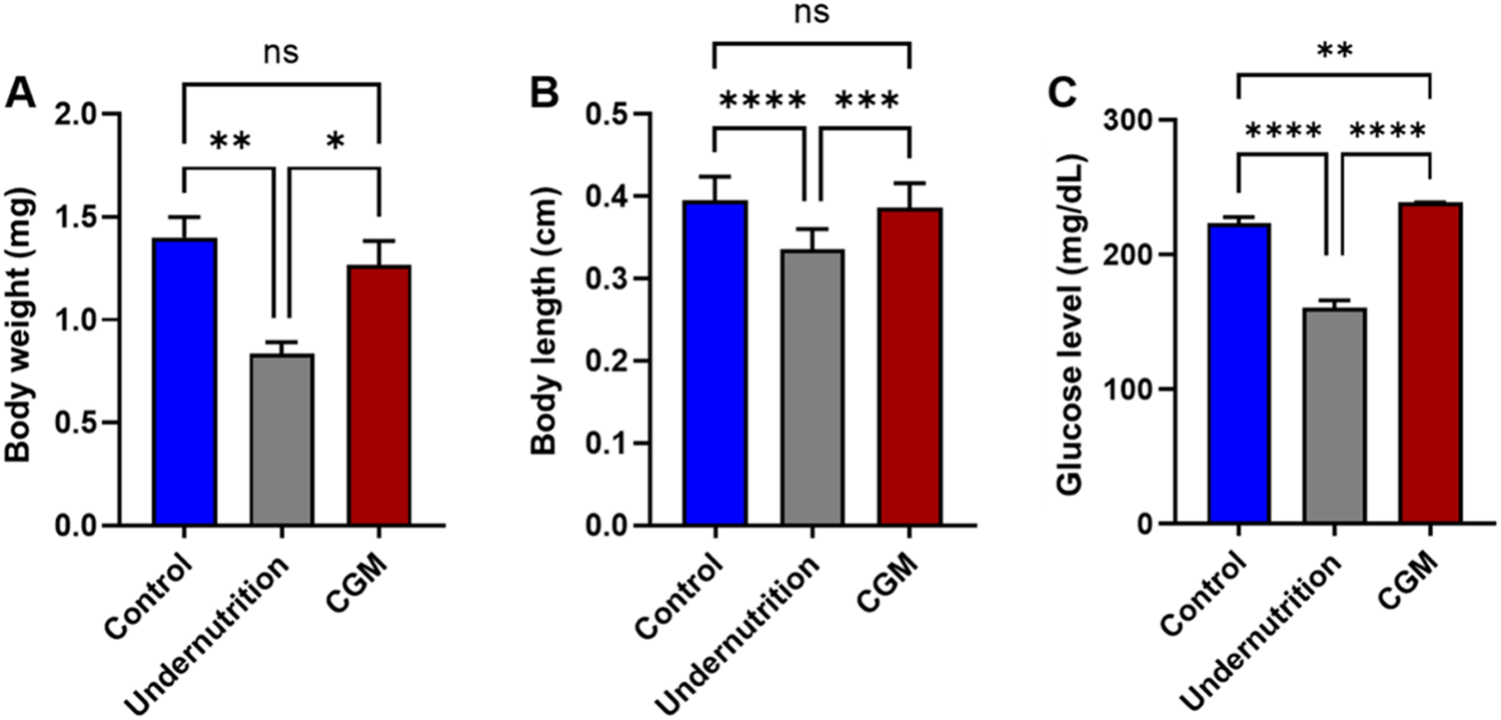
Morphological and metabolic changes in *Drosophila* larvae under catch-up growth conditions. The catch-up growth model (CGM) exhibited significantly elevated glucose levels (C), while no significant differences were observed in body weight (A) or body length (B) compared with the control group. Statistical analysis was performed using one-way ANOVA followed by Tukey’s multiple-comparison test (*n* = 3 independent biological replicates). Data are presented as mean ± SD. ns, not significant; *p < 0.05; **p < 0.01; ***p < 0.001; and ****p < 0.0001.

This study further demonstrated that catch-up growth conditions were associated with elevated glucose levels in larvae (Figure 2C). This increase is consistent with the thrifty energy hypothesis, which proposes that metabolic adaptations following periods of nutrient deprivation enhance glucose conservation within tissues. As a result, circulating glucose in the hemolymph remains elevated to sustain rapid compensatory growth, even in the presence of increased insulin signaling.^30^

### 3.2 Catch-Up Growth Induces a Hyperglycemic Phenotype in Adult Flies

Catch-up growth has been associated with increased susceptibility to metabolic disorders, including hyperglycemia and the subsequent risk of T2DM. To determine whether these effects persist into adulthood in *Drosophila*, we assessed body surface area, glucose levels, and the expression of insulin-related genes (*dilp2* and *dInR*) in adult flies that underwent catch-up growth. Adult flies in the catch-up growth model (CGM) group displayed body surface areas comparable to those of normally fed controls (Figure 3A), indicating the restoration of somatic growth. However, CGM adults exhibited significantly elevated glucose levels (Figure 3B), suggesting persistent metabolic dysregulation. Furthermore, the expression of *dilp2* and *dInR* was markedly increased in CGM adults (Figure 3C–D), consistent with compensatory activation of insulin signaling in response to hyperglycemia. Collectively, these findings indicate that catch-up growth results in sustained metabolic alterations beyond the larval stage, leading to a hyperglycemic phenotype in adult *Drosophila* that recapitulates early features of T2DM.

**Figure 3:**
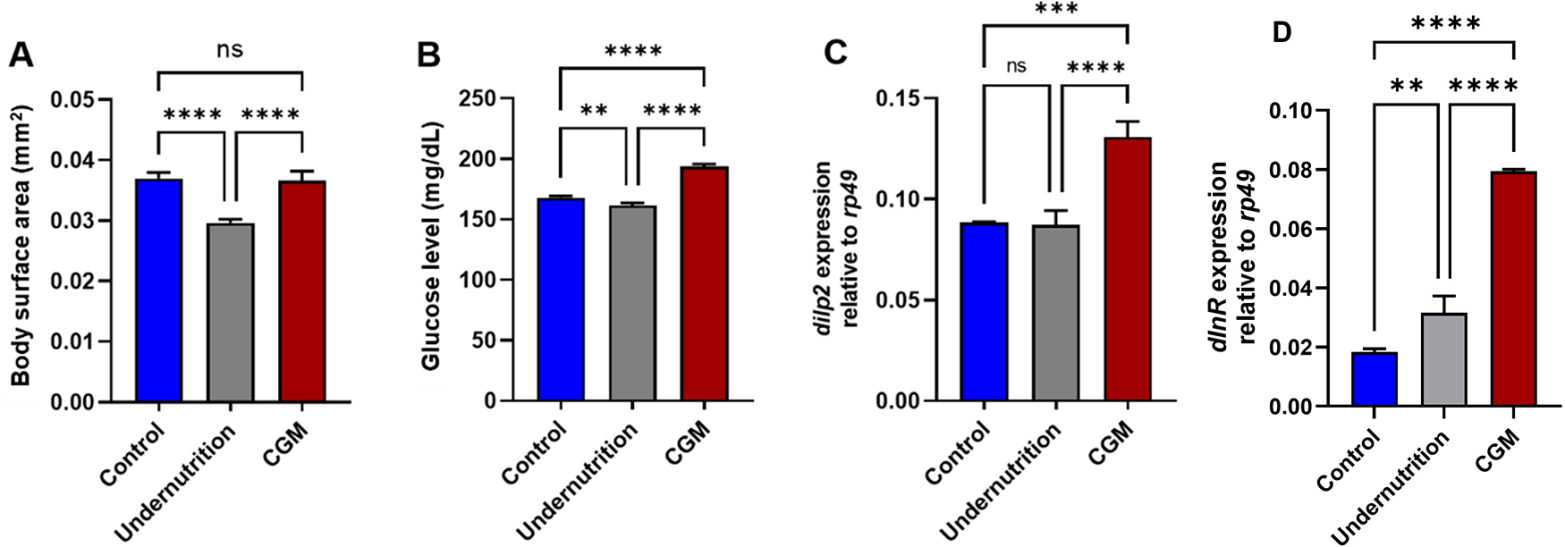
Persistent metabolic alterations associated with catch-up growth in adult *Drosophila*. (A) Body surface area and (B) glucose levels, along with (C, D) *dilp2* and *dInR* expression, showing sustained metabolic alterations beyond the larval stage. Statistical analysis was performed using one-way ANOVA followed by Tukey’s multiple-comparison test (*n* = 3 independent biological replicates). Data are presented as mean ± SD. ns, nonsignificant; **p < 0.01; ***p < 0.001; and ****p < 0.0001.

### 3.3 Catch-Up Growth Increases ROS Levels and Elevates *dPHM* Expression, with No Changes in *dHNF4* Expression

To investigate the mechanisms underlying the type 2 diabetes-like phenotype observed in catch-up growth (CGM) flies, we assessed oxidative stress, ecdysone biosynthesis (*dPHM*), and metabolic regulation (*dHNF4*). ROS levels were significantly elevated in CGM adults compared with those of both normal and undernourished controls (Figure 4A), indicating heightened oxidative stress following nutritional restoration. Increased ROS production is consistent with the elevated metabolic demands associated with compensatory growth and has been linked to mitochondrial dysfunction and impaired insulin signaling.^31^ Excessive ROS can further compromise *insulin-producing cells* (IPCs) and activate stress-responsive kinase pathways, contributing to insulin resistance and reducing Dilp2 secretion even when *dilp2* transcript levels are elevated.^32,33^

**Figure 4:**
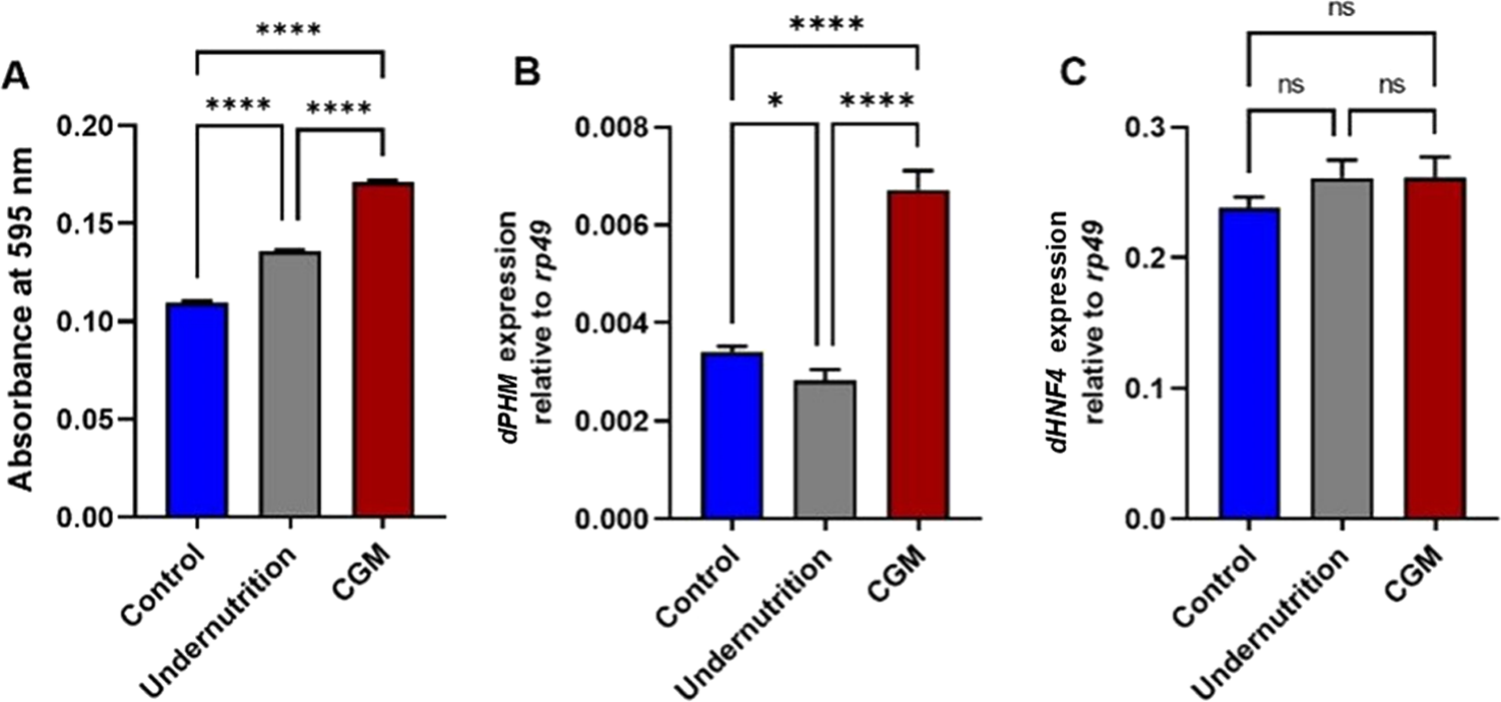
Oxidative stress and hormone-related gene expression in adult *Drosophila* following catch-up growth. (A) ROS levels were measured as absorbance at 595 nm, in control, undernutrition, and catch-up growth model (CGM) flies. (B) Relative expression of *dPHM* and (C) *dHNF4* in adult flies, normalized to *rp49*. Statistical analysis was performed using one-way ANOVA followed by Tukey’s multiple-comparison test (*n* = 3 independent biological replicates). Data are presented as mean ± SD. ns, nonsignificant; *p < 0.05; and ****p < 0.0001.

Expression of *dPHM* was also significantly increased in CGM adults (Figure 4B). Because *dPHM* encodes a key enzyme in the ecdysone biosynthetic pathway, its upregulation suggests enhanced ecdysone activity, potentially reflecting a compensatory response to heightened insulin pathway stimulation. Ecdysone is known to exert an inhibitory feedback on insulin production, which may contribute to the persistent hyperglycemia observed in CGM flies.

To assess whether CGM induces defects resembling maturity-onset diabetes of the young (MODY), we examined the expression of *dHNF4*. Transcript levels of *dHNF4* did not differ among groups (Figure 4C), suggesting that *HNF4* dysregulation during catch-up growth may occur at the post-translational rather than transcriptional level. Because *HNF4* plays a central role in carbohydrate and lipid metabolism, unchanged mRNA levels in the presence of elevated ROS point toward a functional imbalance that mirrors a MODY-like metabolic profile.^34^ ROS has been reported to impair mitochondrial function and suppress *HNF4* activity without altering gene expression, potentially creating a mismatch between insulin-ecdysone signaling and glucose homeostasis. Such a disruption may produce a MODY-like phenotype driven by acquired metabolic stress rather than inherited mutations.

### 3.4 Metabolic and Functional Readouts Associated with T2DM-like Features under Catch-Up Growth Conditions

To further characterize the metabolic and functional consequences of catch-up growth, we next examined additional readouts commonly associated with early T2DM-like alterations in *Drosophila*. In addition to hyperglycemia, dysregulation of lipid storage^35^ and age-dependent decline in locomotor performance have been widely used as complementary indicators of metabolic imbalance and impaired physiological function. Therefore, triglyceride levels and locomotor activity were assessed to provide an integrated view of metabolic status and organismal function under catch-up growth conditions.

Catch-up growth flies exhibited a significant increase in triglyceride (TG) level compared with control flies, indicating altered lipid storage, consistent with a T2DM-like metabolic state (Figure 5A). In contrast, locomotor performance showed an age-dependent decline across all groups with no statistically significant difference between CGM and control flies at day 7 of adulthood (Figure 5B–C). These findings suggest that catch-up growth primarily induces metabolic dysregulation, particularly at the level of lipid homeostasis, while gross locomotor function remains relatively preserved at early adult stages. Such a dissociation between metabolic impairment and overt functional decline has been reported in *Drosophila* models of insulin resistance and diet-induced metabolic dysfunction, where lipid accumulation and hyperglycemia precede more pronounced functional deterioration.^36^

**Figure 5:**
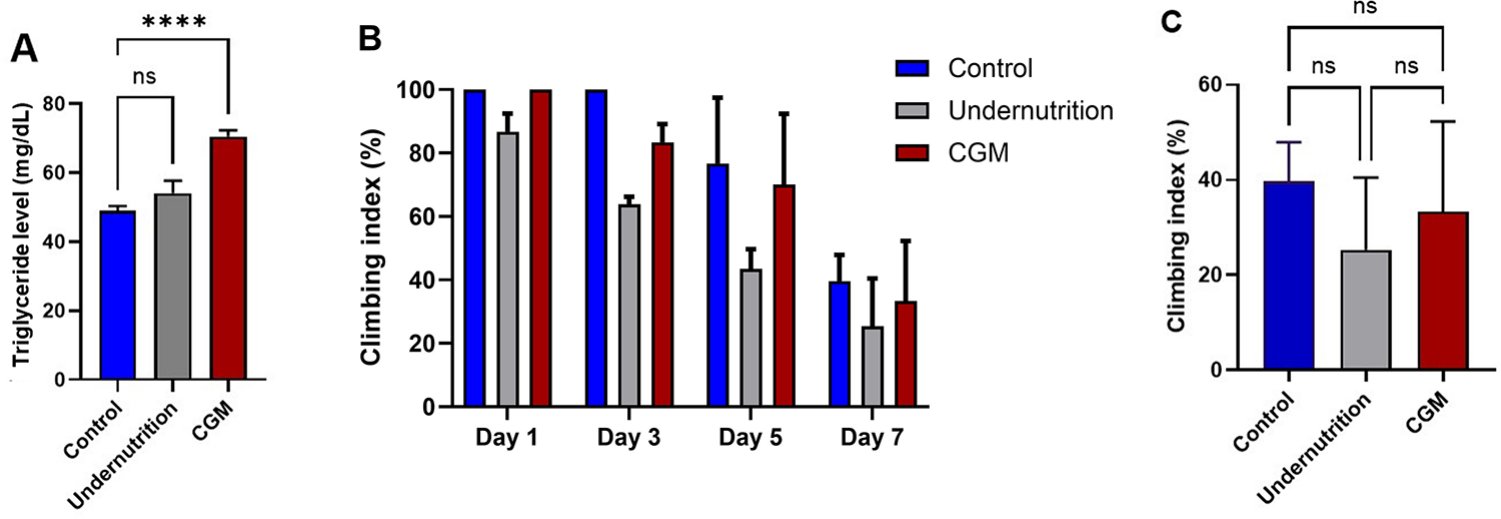
Metabolic and functional readouts associated with T2DM-like features under catch-up growth conditions. (A) Triglyceride levels in adult flies from control, undernutrition, and catch-up growth model (CGM) groups. (B) Age-dependent locomotor performance assessed by a climbing assay on days 1, 3, 5, and 7 of adulthood. (C) Locomotor performance at day 7 of adulthood. Data are presented as mean ± SD (*n* = 3 independent biological replicates). Statistical analysis was performed using one-way ANOVA followed by Tukey’s multiple-comparison test with the control group as reference. ns, nonsignificant; ****p < 0.0001.

### 3.5 Repurposing Clomiphene Citrate to Prevent Catch-Up-Growth-Induced Diabetes Mellitus

Prior to evaluating the metabolic effects of CC in catch-up growth flies, we first assessed its safety across developmental stages. Larvae were exposed to increasing concentrations of CC (0.1–2.5 mM), and the developmental progression was monitored. CC concentrations of 0.1 and 0.5 mM did not significantly affect either the larva-to-pupa transition (Figure 6A) or the pupa-to-adult eclosion rate (Figure 6B), indicating that these concentrations are well tolerated and suitable for subsequent metabolic analyses.

**Figure 6:**
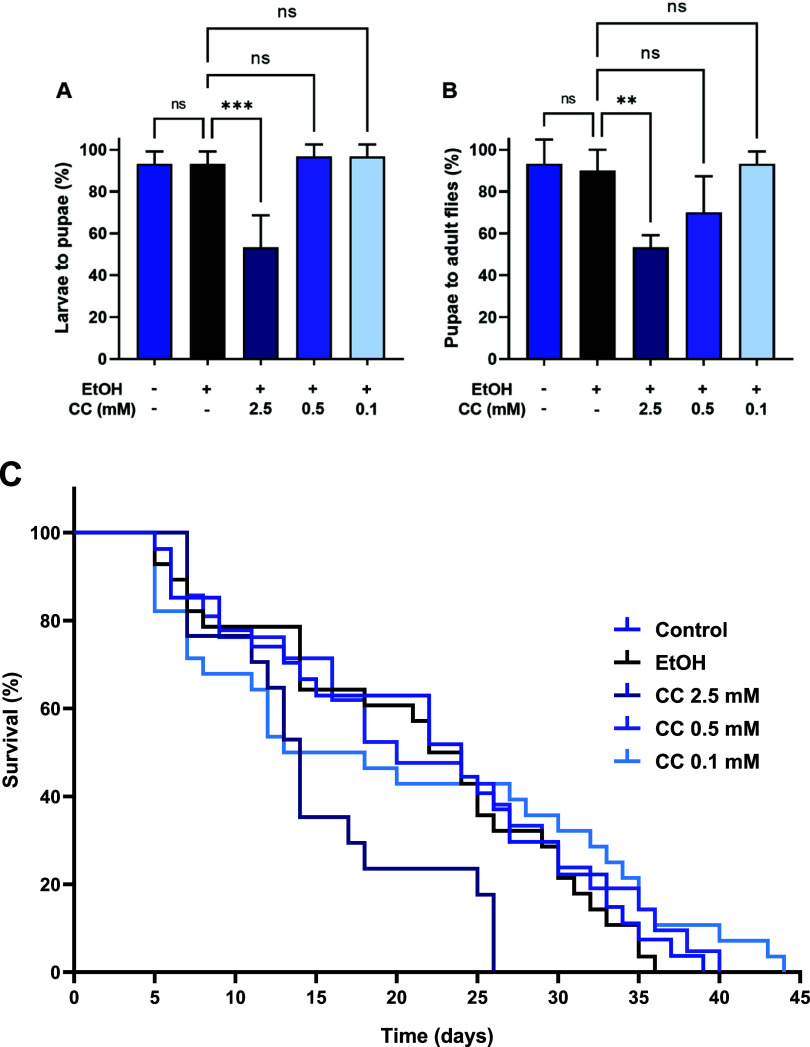
Developmental progression and survival of *Drosophila* under clomiphene citrate (CC) exposure. (A) Larva-to-pupa and (B) pupa-to-adult transition rates under CC exposure. (C) Kaplan–Meier survival curves showing that 0.1 and 0.5 mM CC do not significantly affect survival compared with controls, whereas 2.5 mM CC markedly reduces survival and impairs developmental progression. Data are presented as mean ± SD (*n* = 3 independent biological replicates for A and B). Statistical analysis was performed using one-way ANOVA followed by Tukey’s multiple-comparison test for developmental rates and the log-rank (Mantel-Cox) test for survival analysis. ns, not significant; **p < 0.01; and ***p < 0.001.

Survival analysis further demonstrated that 0.1 and 0.5 mM CC did not reduce lifespan compared with the ethanol vehicle control (Figure 6C). In contrast, exposure to 2.5 mM CC produced clear toxicity, as indicated by impaired developmental progression and significantly reduced adult survival. This adverse effect may be attributable to the limited solubility of CC at higher concentrations in 70% ethanol, which can result in precipitation and inconsistent dietary exposure. Taken together, these findings indicate that 0.5 mM represents the highest nontoxic and physiologically acceptable concentration of CC in *Drosophila* and is therefore appropriate for subsequent evaluation of its metabolic effects in the catch-up growth model.

The physiological relevance of the CC concentrations employed in this study should be interpreted cautiously within a translational framework. In *Drosophila*, pharmacological compounds are delivered through the diet, and systemic exposure is influenced by feeding behavior, intestinal absorption, metabolic turnover, and bioavailability constraints. As a result, dietary concentrations in the micromolar to millimolar range are often necessary to achieve comparatively modest internal exposure. These concentrations therefore cannot be directly extrapolated to, or equated with, plasma levels observed in mammalian systems.

Importantly, the CC concentrations evaluated (0.1–0.5 mM) were subtoxic and did not produce overt developmental abnormalities, increased mortality, or impaired locomotor performance. Rather than eliciting signs of generalized stress, these doses were associated with reduced oxidative stress and improved metabolic parameters, suggesting that the observed effects are unlikely to result from nonspecific toxicity. Although direct quantification of CC bioavailability and metabolic stability in *Drosophila* was beyond the scope of the present study, the absence of stress-related phenotypes supports the interpretation that CC exerts dose-tolerated metabolic modulation rather than off-target toxic effects. Accordingly, the reported concentrations correspond to dietary exposure levels and do not represent directly measured internal tissue concentrations.

Accordingly, the present findings should be interpreted as providing mechanistic and biological proof-of-concept for CC-mediated metabolic modulation in the context of catch-up growth rather than as a direct predictor of clinically relevant human dosing. Future investigations incorporating pharmacokinetic characterization and validation in mammalian models will be necessary to more precisely establish the translational relevance of CC exposure levels.

### 3.6 Validation of the Catch-Up Growth Model Using Metformin as a Positive Control

To confirm that the catch-up growth model is responsive to established antihyperglycemic interventions, metformin was included as a positive control. Metformin is widely used as a glucose-lowering agent across experimental systems and therefore provides a functional benchmark to assess whether the observed hyperglycemic phenotype in CGM flies can be pharmacologically modulated. In this context, metformin was administered to CGM flies, and glucose levels were measured as the primary metabolic end point to validate model sensitivity.

Metformin was included as a positive control to assess the metabolic responsiveness of the catch-up growth model. As expected, CGM flies displayed significantly elevated glucose levels (Figure 7A) compared with the controls, confirming the presence of a hyperglycemic phenotype. Metformin treatment markedly reduced glucose levels relative to CGM, indicating that the model is responsive to a well-established glucose-lowering intervention.

**Figure 7:**
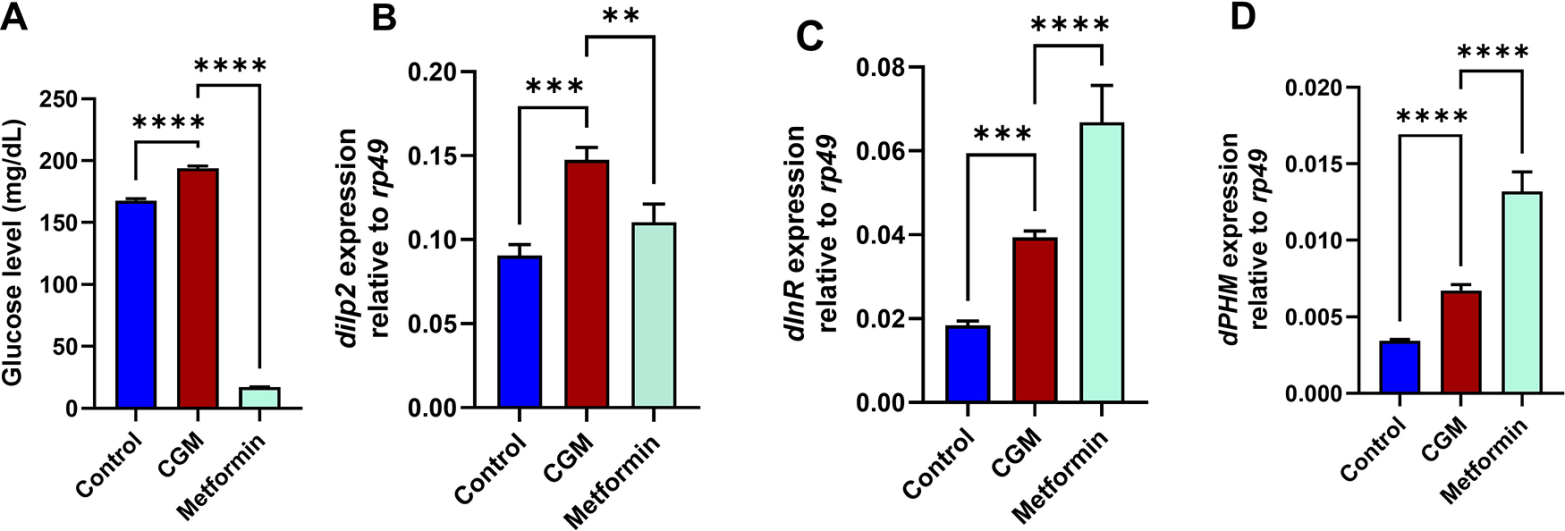
Validation of the catch-up growth model using metformin as a positive control. (A) Glucose levels in adult flies from control, catch-up growth model (CGM), and metformin-treated groups. (B–D) Relative mRNA expression normalized to *rp49*: (B) *dilp2*, (C) *dInR*, and (D) *dPHM*. Data are presented as mean ± SD (*n* = 3 independent biological replicates). Statistical analysis was performed using one-way ANOVA followed by Tukey’s multiple-comparison test. **p < 0.01; ***p < 0.001; and ****p < 0.0001.

At the transcriptional level (Figure 7B–D), CGM flies exhibited altered expression of insulin- and hormone-related genes (*dilp2*, *dInR*, and *dPHM*). Metformin treatment modulated these transcripts, although the direction and magnitude of change varied across markers. These results suggest that the CGM phenotype reflects a metabolically perturbed state that can be pharmacologically modulated, rather than representing nonspecific metabolic stress.

### 3.7 Clomiphene Citrate Does Not Alter Body Weight in Catch-Up Growth Larvae but Modulates Insulin Signaling Pathways

To evaluate the metabolic effects of CC in the catch-up growth model, we assessed larval body weight, insulin-related gene expression, and adult glucose levels. CC administration at 0.1 or 0.5 mM did not significantly affect the body weight of late third-instar larvae (Figure 8A), indicating that CC does not interfere with somatic growth during the compensatory phase. In contrast, CC produced clear effects on insulin signaling. Treatment with 0.1 mM CC significantly reduced *dilp2* and *dInR* transcript levels in catch-up growth adults (Figure 8B–C), accompanied by a marked decrease in glucose levels (Figure 8D). These findings indicate that CC attenuates the hyperactivation of insulin-like signaling typically observed in catch-up growth flies and helps restore glucose homeostasis.

**Figure 8:**
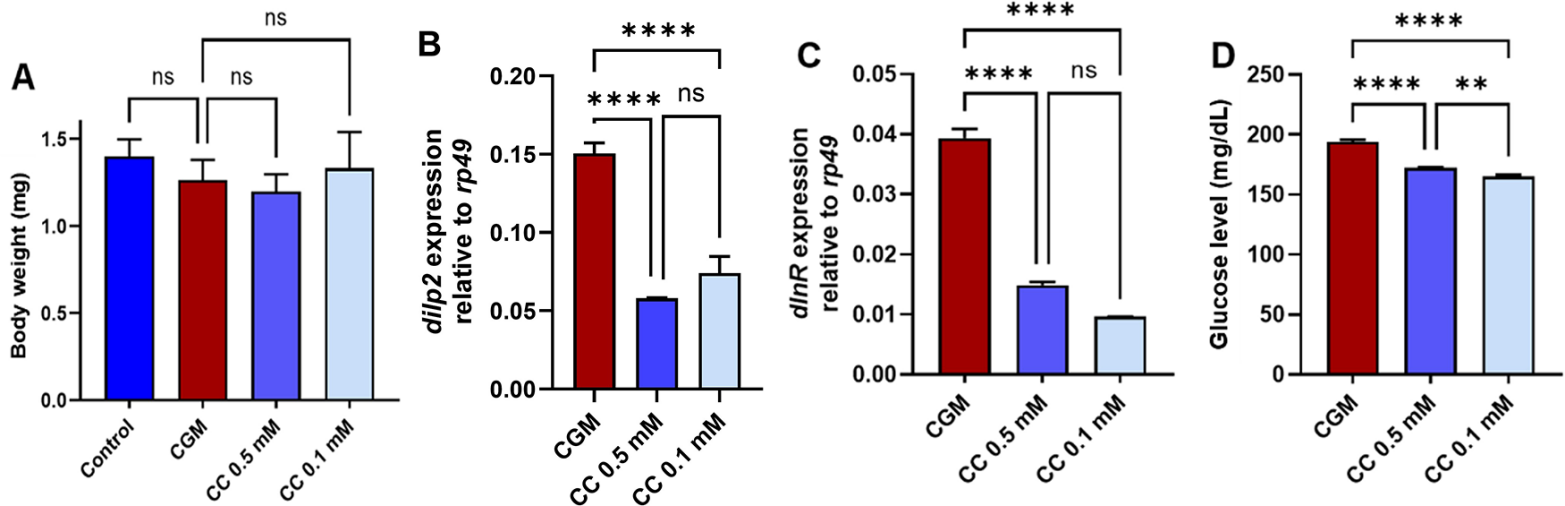
Metabolic effects of clomiphene citrate (CC) in the catch-up growth model (CGM). (A) CC treatment did not significantly alter larval body weight, indicating no overt effect on somatic growth. (B, C) CC supplementation was associated with reduced expression of insulin-related genes *dilp2* and *dInR* in adult flies under catch-up growth conditions. (D) CC treatment, particularly at 0.1 mM, significantly lowered glucose levels compared with untreated catch-up growth flies, consistent with improved glucose homeostasis. Data are presented as mean ± SD (*n* = 3 independent biological replicates). Statistical analysis was performed using one-way ANOVA followed by Tukey’s multiple-comparison test. ns, not significant; **p < 0.01; and ****p < 0.0001.

Although CC is widely characterized as a selective estrogen receptor modulator (SERM) in mammals, *Drosophila* lacks canonical estrogen receptors. Therefore, the metabolic effects observed in this model are unlikely to be mediated by classical estrogen signaling pathways. Instead, metabolic regulation in *Drosophila* depends on pathways involving insulin, ecdysone, nutrient sensors, and nuclear receptors such as dERR, which plays a critical role in coordinating glucose utilization and lipogenesis during early adulthood.^37^ In this context, the observed effects of CC are consistent with modulation of insulin-associated and nuclear receptor-linked metabolic pathways rather than estrogenic mechanisms. Notably, CC treatment was associated with a reversal of key metabolic alterations induced by catch-up growth, including elevated glucose levels and increased expression of *dilp2* and *dPHM*, while growth-related parameters remained unchanged. These findings indicate that CC preferentially affects metabolic regulatory readouts under catch-up growth conditions without overtly altering developmental growth control. Accordingly, the improvement in glucose homeostasis observed in CC-treated flies supports the interpretation that CC is associated with the modulation of insulin-related metabolic regulation in a physiological context resembling early-stage T2DM-like dysfunction, while the precise molecular targets remain to be elucidated.

### 3.8 Clomiphene Citrate Decreases ROS Levels and Reduces the Expression of *dHNF4* and *dPHM*

To determine whether CC modulates oxidative and hormonal pathways in the catch-up growth model, we examined ROS levels and the expression of *dHNF4* and *dPHM* in young adult flies. CC treatment markedly reduced ROS levels compared with untreated catch-up-growth groups (Figure 9A), indicating attenuation of oxidative stress following treatment. Lower ROS levels may contribute to improved insulin sensitivity by limiting the activation of stress-responsive pathways.

**Figure 9:**
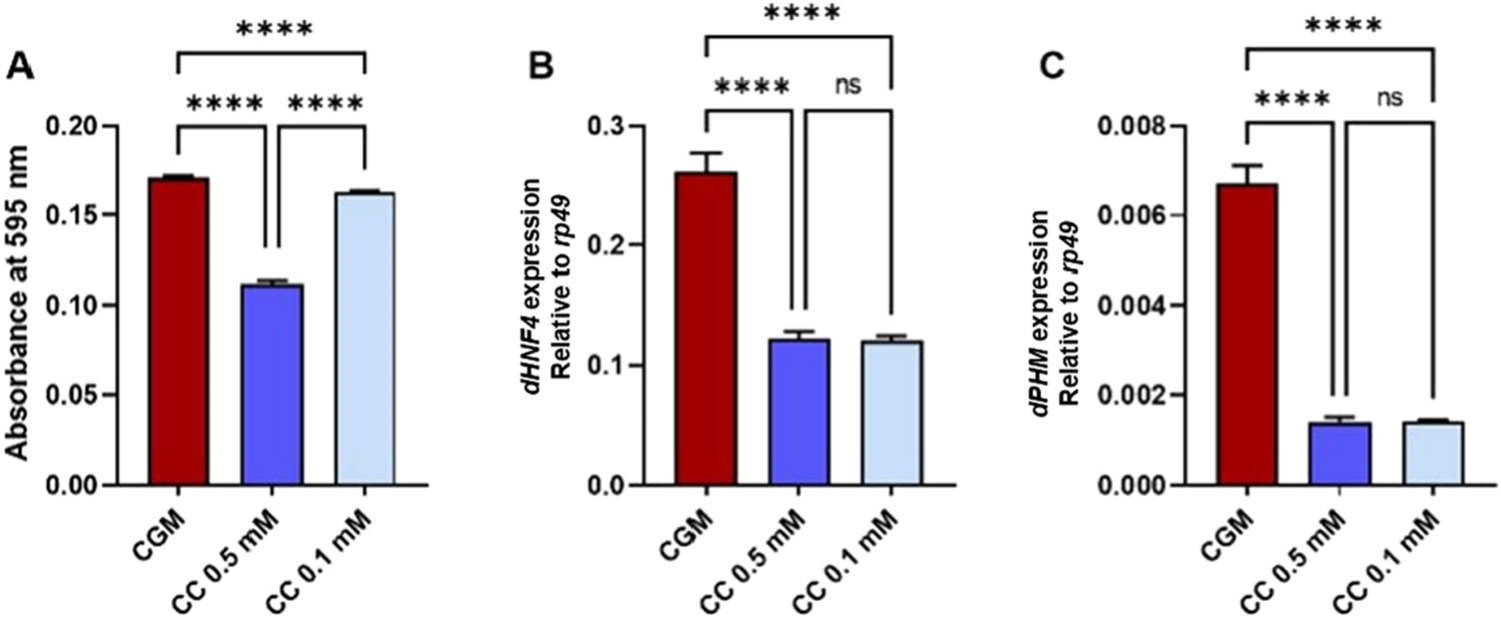
Effects of clomiphene citrate (CC) on oxidative stress and hormone-related gene expression in the catch-up growth model. (A) CC treatment was associated with a significant reduction in reactive oxygen species (ROS) levels, as measured by absorbance at 595 nm. (B, C) CC supplementation was associated with reduced expression of *dHNF4* and *dPHM* relative to untreated catch-up growth flies, indicating modulation of oxidative and hormone-linked transcriptional pathways. Data are presented as mean ± SD (*n* = 3 independent biological replicates). Statistical analysis was performed using one-way ANOVA followed by Tukey’s multiple-comparison test. ns, not significant; ****p < 0.0001.

CC also significantly reduced the levels of expression of *dHNF4* (Figure 9B) and *dPHM* (Figure 9C). Because *dPHM* encodes a key enzyme in the ecdysone biosynthetic pathway, its downregulation suggests the inhibition of ecdysone-dependent responses that are typically elevated during compensatory growth. The decrease in the level of *dHNF4* expression further implies the modulation of metabolic programming governing lipid and carbohydrate homeostasis. Given the central role of *dHNF4* in glucose regulation, reduced *dHNF4* levels may help counteract the MODY-like metabolic imbalance associated with catch-up growth. Taken together, the reduction in ROS levels and the normalization of *dPHM* and *dHNF4* expression indicate that CC acts through both oxidative and nuclear-receptor-dependent pathways, complementing its effects on insulin signaling. These findings suggest that CC functions as a metabolic modulator capable of mitigating the maladaptive metabolic reprogramming characteristic of catch-up growth.

### 3.9 In Silico Docking and Molecular Dynamics Reveal Differential Binding and Stability of Clomiphene Citrate Across Metabolic Targets

Molecular docking was performed to evaluate the interaction of CC with three key metabolic targets implicated in the catch-up growth phenotype: dilp2, EcR, and HNF. Docking results showed that CC had the highest predicted binding affinity for EcR (Δ*G* = −7.9 kcal·mol^–1^), followed by HNF (Δ*G* = −5.0 kcal·mol^–1^) and dilp2 (Δ*G* = −3.7 kcal·mol^–1^). Structural visualization confirmed that CC occupied the active-site cavities of all three proteins, forming hydrogen bonds and hydrophobic interactions indicative of stable ligand-protein association (Figure 10).

**Figure 10:**
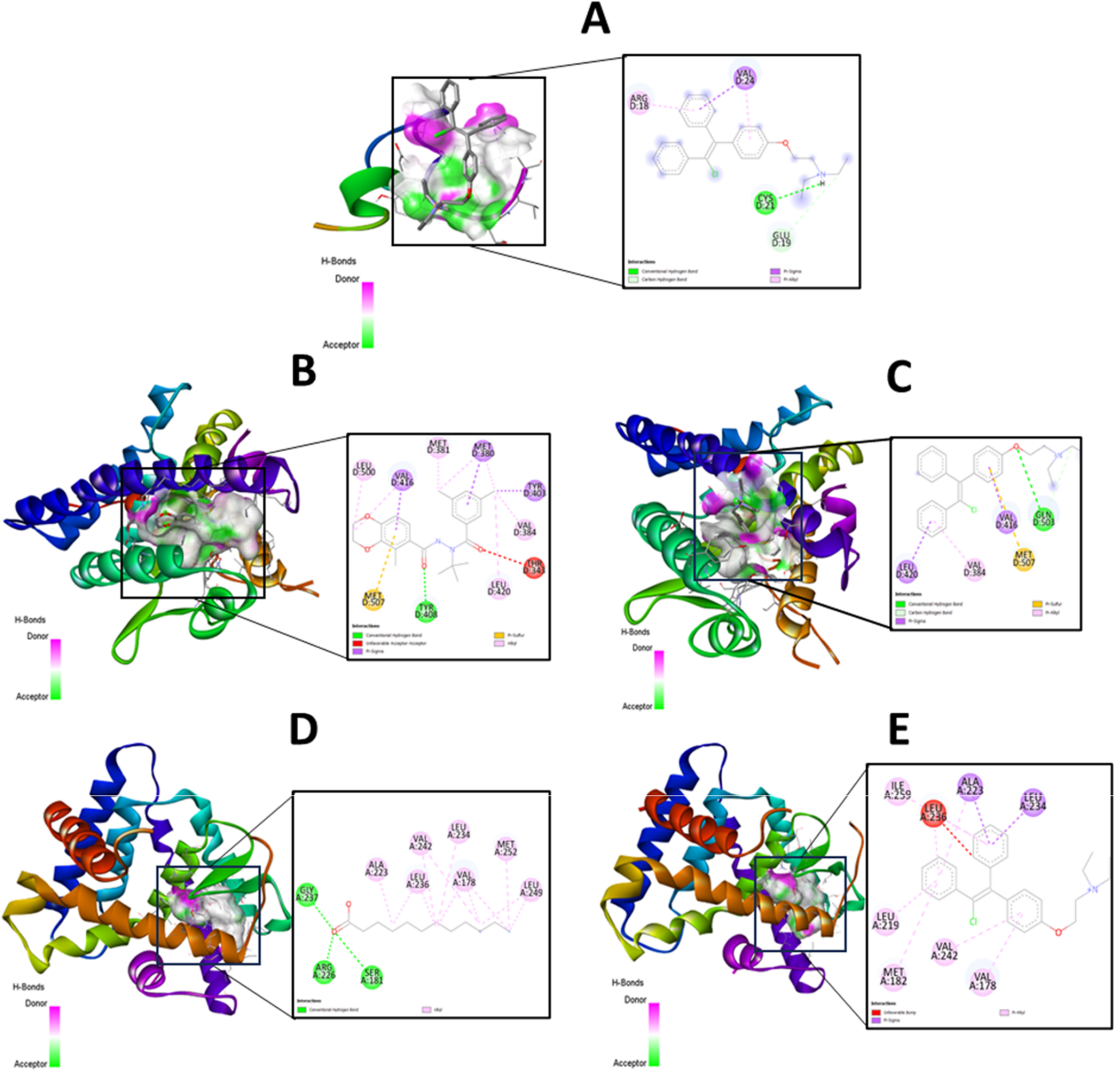
Three-dimensional (3D) visualization of the predicted binding orientation of clomiphene within the active sites of (A) *Drosophila* insulin-like peptide 2 (Dilp2) – clomiphene interaction, (B) ecdysone receptor (EcR) – native ligand interaction, (C) ecdysone receptor (EcR) – clomiphene interaction, (D) hepatocyte nuclear factor (HNF) – native ligand interaction, (E) hepatocyte nuclear factor (HNF) – clomiphene interaction obtained from molecular docking simulations using AutoDock Vina.

To further characterize the stability and dynamic behavior of these complexes, 100 ns MD simulations were conducted. In the dilp2-clomiphene complex, both the radius of gyration (*R*_g_) and RMSD stabilized after approximately 25 ns, suggesting an initial structural adjustment followed by convergence to a compact, stable conformation (Figure 11A–C). RMSF analysis revealed moderate flexibility at the terminal residues, a pattern consistent with known dilp2 dynamics.

**Figure 11:**
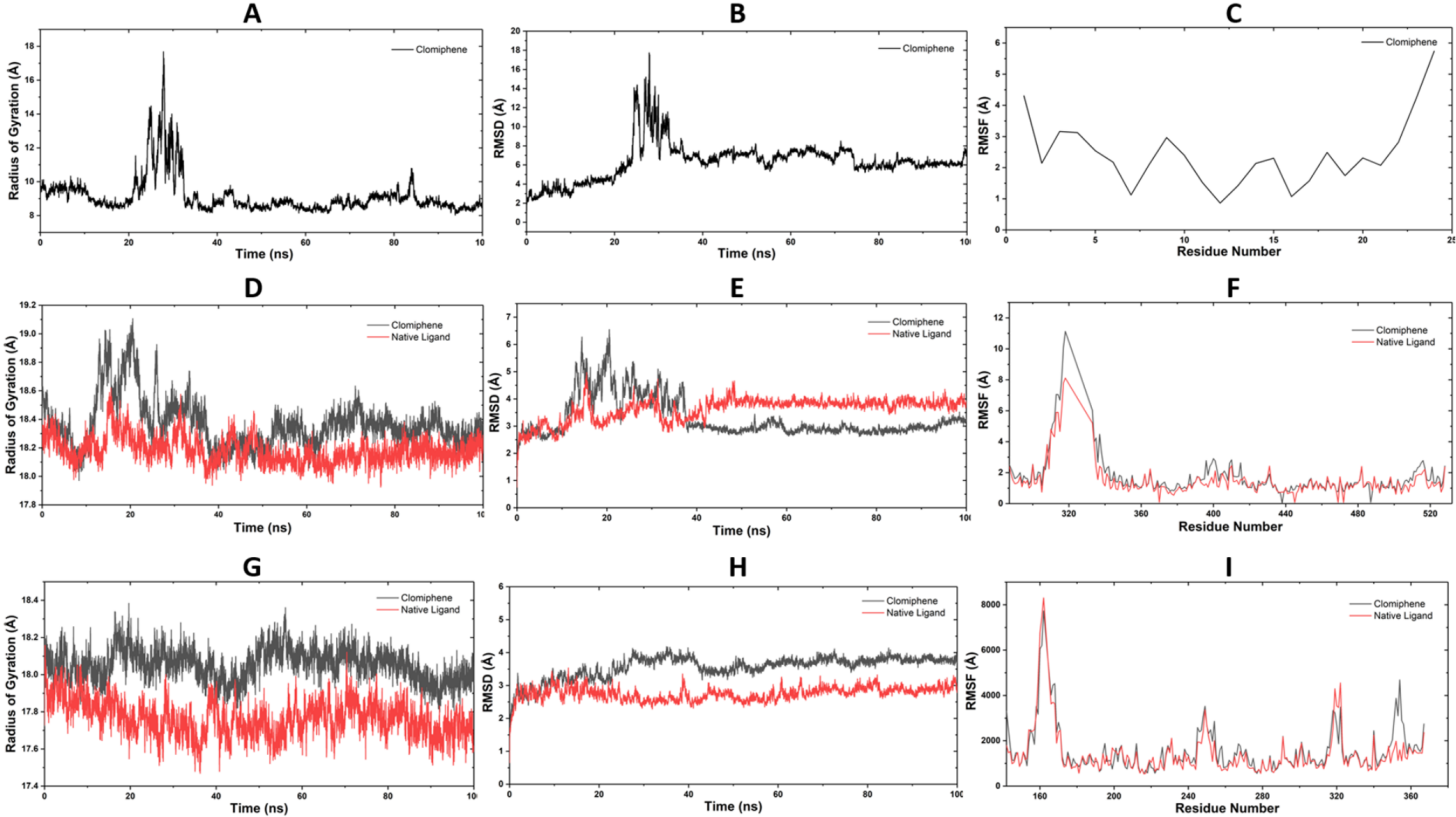
Molecular dynamics characterization of clomiphene-bound systems is presented across three target proteins. (A–C) Temporal evolution of key structural descriptors for the dilp2 complex, including global compactness (*R*_g_), backbone stability (RMSD), and residue-resolved flexibility (RMSF). (D–F) Comparative assessment of Clomiphene (black) and native ligand (red) behavior within the EcR receptor, revealing distinct differences in conformational stability and local fluctuations. Similarly, (G–I) Dynamic disparities between Clomiphene and the native ligand in the HNF system. Together, *R*_g_, RMSD, and RMSF analyses delineate ligand-induced alterations in both global protein folding pattern and structural flexibility across the targets, reflecting distinct conformational adaptations throughout the 100 ns molecular dynamics simulation.

In the EcR system, clomiphene binding resulted in higher and more variable RMSD and *R*_g_ values compared with the native ligand (Figure 11D–F), indicating increased conformational mobility. RMSF profiles showed local fluctuations near the ligand-binding pocket, suggesting mild destabilization associated with clomiphene occupancy. For the HNF complex, clomiphene induced consistently elevated *R*_g_ and RMSD values relative to those of its native ligand (Figure 11G–I), reflecting a more flexible and expanded binding configuration.

Overall, the MD simulations indicate that clomiphene can form stable interactions with all three targets but with distinct dynamic signatures: relatively stable binding to dilp2, moderate structural adaptability in EcR, and greater conformational flexibility in HNF. These computational findings complement the docking results and provide a structural basis for hypothesizing potential molecular targets of clomiphene; however, they do not establish a direct causal link to the physiological effects observed in the catch-up growth model.

It is, therefore, important to distinguish between the levels of evidence provided by the computational and experimental components of this study. Docking and molecular dynamics simulations predict potential direct molecular interactions between CC and selected nuclear receptors but do not constitute direct evidence of receptor engagement or activation in vivo, nor do they establish causality with downstream metabolic effects. In contrast, the observed changes in glucose homeostasis, oxidative balance, and transcriptional markers represent indirect physiological outcomes that likely arise from integrated systemic regulation rather than from a one-to-one consequence of receptor binding. Accordingly, the docking and MD analyses should be interpreted as hypothesis-generating, while the in vivo phenotypes provide functional evidence of metabolic modulation without defining the precise molecular point of action.

### 3.10 Linking Physiological Effects of Clomiphene Citrate to Its Predicted Molecular Interactions

The in vivo experiments demonstrated that CC, at subtoxic concentrations (0.1–0.5 mM), effectively lowered glucose levels in catch-up growth flies and normalized the expression of *dilp2* and *dPHM*, accompanied by reduced ROS levels and modulation of *dHNF4* expression (Figures 6 and 7). These combined physiological and molecular outcomes indicate that CC modulates glucose homeostasis and oxidative balance in the *Drosophila* catch-up growth model, consistent with a role in metabolic programming rather than direct insulin-mimetic activity.

To strengthen phenotypic validation beyond hyperglycemia and transcriptional markers, we further examined triglyceride (TG) levels as an indicator of the lipid metabolic status. Catch-up growth flies exhibited a significant increase in TG levels compared to controls, indicating dysregulated lipid storage. Altered lipid homeostasis is a conserved hallmark of insulin resistance and early T2DM-like metabolic states in *Drosophila* and has been widely used as a complementary readout alongside hyperglycemia and impaired insulin signaling.^35,36^

In contrast, locomotor performance assessed by climbing assays did not show a significant impairment in catch-up growth flies at early adult stages. Preservation of gross locomotor function despite clear metabolic alterations argues against generalized physiological decline, toxicity, and acute stress responses. Instead, this dissociation between metabolic dysregulation and functional impairment is consistent with early-stage insulin resistance models in *Drosophila*, in which hyperglycemia and lipid accumulation precede overt behavioral or neuromuscular deficits.

Together, the combination of sustained hyperglycemia, altered insulin-related transcription, oxidative stress, and dysregulated lipid storage, while maintaining relatively preserved locomotor function, supports the interpretation that the catch-up growth model represents an early-stage or partial T2DM-like metabolic phenotype rather than a fully developed diabetic state or nonspecific metabolic stress. This framing aligns with established *Drosophila* models of diet-induced metabolic dysfunction and appropriately reflects the biological scope of a nonmammalian system.

The in silico docking analysis supports these observations by predicting that clomiphene interacts most strongly with EcR (Δ*G* = −7.9 kcal·mol^–1^), followed by HNF (Δ*G* = −5.0 kcal·mol^–1^), whereas its affinity for dilp2 is comparatively lower (Δ*G* = −3.7 kcal·mol^–1^). This binding hierarchy (EcR > HNF > Dilp2) aligns with the interpretation that CC’s metabolic effects are likely mediated through nuclear receptors or transcription factors rather than via direct engagement with the insulin-like peptide itself.

Based on in silico predictions, clomiphene binding within the EcR ligand-binding domain may influence ecdysone-dependent signaling, consistent with the reduced *dPHM* expression observed in vivo. The relatively weak predicted interaction with Dilp2 further supports the interpretation that CC’s effects on insulin-like signaling occur indirectly rather than through direct engagement with insulin-like peptides. While the present findings implicate EcR- and HNF-related pathways in the metabolic effects of CC, the available data do not allow definitive classification of CC as an agonist, antagonist, or allosteric modulator of these nuclear receptors. In the absence of direct receptor-binding assays or transcriptional reporter analyses, such pharmacological distinctions remain inferential.

Functionally, the observed downregulation of *dPHM* expression and attenuation of ecdysone-associated responses in CC-treated flies is more consistent with partial inhibition or functional antagonism of EcR-dependent signaling rather than receptor activation. However, indirect or context-dependent modulation-potentially mediated through altered coregulator recruitment, hormonal crosstalk, or secondary metabolic feedback-cannot be excluded. Similarly, changes in *dHNF4* expression are indicative of the modulation of HNF-linked metabolic transcriptional programs, without distinguishing between direct receptor antagonism and downstream adaptive effects. Collectively, these observations support the interpretation that CC acts as a context-dependent modulator of nuclear receptor-associated metabolic pathways, rather than a classical agonist or antagonist in the strict pharmacological sense.

Interpretation of the *dHNF4* results requires a context-dependent perspective. In untreated CGM flies, *dHNF4* expression was unchanged, indicating that altered *dHNF4* transcription is not a primary driver of the CGM phenotype. In contrast, CC treatment was associated with reduced *dHNF4* expression, coinciding with improvements in glucose homeostasis, oxidative balance, and insulin-related transcriptional markers.

Importantly, reduced *dHNF4* expression should not be interpreted as intrinsically beneficial. Rather, in the context of improved metabolic status, this change likely reflects adaptive rebalancing of metabolic transcriptional programs rather than pathological suppression. The absence of growth impairment or metabolic deterioration in CC-treated flies argues against oversuppression of *dHNF4* activity. Nevertheless, the data presented here do not allow definitive discrimination between normalization and partial suppression of *dHNF4* signaling, which we acknowledge as a limitation requiring further investigation.

Some discrepancies between predicted binding energies and the magnitude of in vivo responses may reflect pharmacokinetic factors such as compound bioavailability, tissue distribution, formation of active metabolites, or uncharacterized off-target interactions. To further clarify the mechanistic basis of CC activity, additional experimental validation, such as in vitro binding assays for EcR and HNF, transcriptional reporter assays, basic pharmacokinetic characterization, and extended MD simulations with MM-PBSA energy decomposition, is recommended.

It is important to emphasize that the present study does not support the direct clinical translation of CC for metabolic disease. Instead, CC is employed as a pharmacological tool to probe conserved metabolic regulatory pathways under catch-up growth conditions in a genetically tractable invertebrate model. Given the absence of canonical estrogen receptor signaling in *Drosophila*, the observed effects are unlikely to reflect CC’s known endocrine mechanisms in mammals. Accordingly, these findings should be interpreted as hypothesis-generating, with future validation in mammalian models required to assess translational relevance.

## 4 Conclusion

This study establishes *D. melanogaster* as a tractable and biologically relevant model for investigating catch-up-growth-associated metabolic disturbances that resemble early-stage or partial T2DM-like features. The model recapitulated key metabolic alterations, including sustained hyperglycemia, increased oxidative stress, and dysregulation of insulin- and hormone-related transcripts (*dilp2, dInR*, *dPHM*, and *dHNF4*), supporting its utility for studying metabolic programming under catch-up growth conditions.

Within this framework, CC was used as a pharmacological probe to explore conserved metabolic regulatory pathways. At subtoxic concentrations, CC was associated with reduced oxidative stress, improved glucose homeostasis, and modulation of hormone-linked transcriptional responses. Given the absence of canonical estrogen receptor signaling in *Drosophila*, these effects are unlikely to reflect CC’s known endocrine mechanisms in mammals and instead point to context-dependent modulation of conserved nuclear receptor-associated metabolic pathways.

Complementary molecular docking and molecular dynamics simulations provided structural insights that are hypothesis-generating, suggesting potential interactions between CC and key metabolic regulators while not establishing direct causal mechanisms. Accordingly, the present findings should not be interpreted as evidence supporting direct therapeutic repurposing of CC for metabolic disease.

Overall, this work highlights *Drosophila* as a powerful experimental system for dissecting early metabolic consequences of catch-up growth and for mechanistic exploration of candidate compounds. Further validation in mammalian models will be required to assess the translational relevance and clinical applicability.

## Supplementary Material



## Notes

Caloric content and macronutrient composition of experimental diets determined by proximate analysis (Table S1) (PDF)
