# Supplementary material for: Clomiphene Citrate Ameliorates Hyperglycemic Phenotype Induced by Catch-Up Growth in Stunting-Like Drosophila
Source: ACS Omega. 2026 Apr 7;11(15):22888–901. doi: 10.1021/acsomega.5c12117 (PMC13103799; doi:10.1021/acsomega.5c12117)
Supplement: Supplementary file 1 [file ao5c12117_si_001.pdf]

Supporting Information

Clomiphene Citrate Ameliorates Hyperglycemic Phenotype Induced by Catch-Up Growth in Stunting-Like *Drosophila*

Hendra Stevani[1,2], Habibie Habibie[3], Muhammad Rayza Azmin[4], Nadila Pratiwi Latada[4], Asbah Asbah[3,4], Widya Hardiyanti[4,5], Mukarram Mudjahid[3,4], Muh. Arfandy Gunawan[6], Firzan Nainu[3,4*]

[1] Doctoral Program in Pharmacy, Faculty of Pharmacy, Hasanuddin University, Tamalanrea, Makassar, Indonesia 90245

[2] Program Study of Bachelor of Applied Pharmacy, Department of Pharmacy, Health Polytechnic of Makassar, Ministry of Health of the Republic of Indonesia, Baji Gau, Makassar, Indonesia 90134

[3] Department of Pharmacy, Faculty of Pharmacy, Hasanuddin University, Tamalanrea, Makassar, Indonesia 90245

[4] Unhas Fly Research Group, Faculty of Pharmacy, Hasanuddin University, Tamalanrea, Makassar, Indonesia 90245

[5] Program Study of Pharmacy, Faculty of Medicine and Health Sciences, Universitas Muhammadiyah Makassar, Makassar, Indonesia 90221

[6] Chemical Biology and Medicinal Chemistry Research Group, Faculty of Pharmacy, Hasanuddin University, Makassar, Indonesia 90245

***Corresponding author**:
Firzan Nainu

Department of Pharmacy, Faculty of Pharmacy, Hasanuddin University, Tamalanrea, Makassar, Indonesia 90245

E-mail: firzannainu@unhas.ac.id

Table S1. Caloric content and macronutrient composition of the experimental diets determined by proximate analysis.

Parameter	Low-calorie diet	Standard diet	Method
Energy (kcal/100 g)	18.69	66.4	Proximate analysis
Carbohydrate (%)	1.84	7.56	Proximate analysis
Protein (%)	2.27	3.82	Proximate analysis
Lipid (%)	0.25	2.32	Proximate analysis
